# Capacity and kinetics of zearalenone adsorption by *Geotrichum candidum* LG-8 and its dried fragments in solution

**DOI:** 10.3389/fnut.2023.1338454

**Published:** 2024-01-11

**Authors:** Fengping Jiao, Xianping Cui, Shujin Shi, Guozheng Jiang, Mingsheng Dong, Ling Meng

**Affiliations:** ^1^School of Public Health, Shandong First Medical University and Shandong Academy of Medical Sciences, Jinan, China; ^2^Division of Hepatobiliary and Pancreatic Surgery, Affiliated Provincial Hospital, Shandong First Medical University and Shandong Academy of Medical Sciences, Jinan, China; ^3^Yantai Hengyuan Bioengineering Co., Ltd., Yantai, China; ^4^College of Food Science and Technology, Nanjing Agricultural University, Nanjing, China

**Keywords:** ZEN, adsorption, drying mode, solution, kinetics

## Abstract

The application of LG-8 and its dry fragments as zearalenone (ZEN) adsorbents was investigated. The study showed that *Geotrichum candidum* LG-8 and its fragments dried at 55°C or through lyophilization are able to adsorb around 80% of ZEN. However, besides in water and 55°C-drying conditions, SEM indicated that higher 90% of ZEN binding tended to occur when cell walls of fragments were intact with less adhesion among themselves. Notably, ZEN/LG-8 fragments complexes were quite stable, as only 1.262% and 1.969% of ZEN were released after successive pH treatments for 4 h and 5 min. The kinetic data signified that adsorption of ZEN onto LG-8 fragments followed well the pseudo-first-order kinetic model. Isotherm calculations showed Langmuir model was favourable and monolayer adsorption of ZEN occurred at functional binding sites on fragments surface. Therefore, we conclude that it can be an alternative biosorbent to treat water contained with ZEN, since LG-8 is low-cost biomass and its fragments have a considerable high biosorption capacity avoiding impacting final product quality and immunodeficient patients.

## 1 Introduction

The contamination of foods with mycotoxins has been a worldwide significant medical and food safety problem (1). During food processing and storage, some fungal species thrived produce complex toxins (1, 2). These complex molecules are highly resistant against physical and chemical impact, therefore, they tend to remain in food and get easily into human body (2). Mycotoxins are hazardous for human health that causing serious economic losses in food industry (2, 3). Zearalenone (ZEN) is one of the most common and dangerous mycotoxins. ZEN, known as F-toxin, is a highly toxic secondary metabolite produced by *Gibberella* (2, 4). It is commonly found in corn, sorghum, wheat, and their byproducts (1, 2). In 1966, Perrin et al. (4) identified ZEN as a dihydroxybenzoate. The ester bonds of dihydroxybenzoates can be cleaved under alkaline conditions and regenerated when the pH is decreased (5). ZEN poses a significant health risk to humans and animals, because it is challenging to degrade and can lead to various adverse effects. Besides immunotoxicity and cytotoxicity, ZEN exerts a potent estrogen-like effect by competitively binding to estrogen receptor in cell membranes, potentially causing reproductive toxicity (6, 7). These threats underscore the need to address the presence of ZEN in food and feed products to mitigate its harmful effects. Elimination of this mycotoxin has been carried out mainly through two ways of policy and technology.

In the way of policy, international agencies have established strict limits on ZEN levels in corn and other grains. In China, food hygiene standards dictate that ZEN content in edible wheat should not surpass 5.0 μg/kg, while the limit for corn is set at 20 μg/kg (8, 9). Following the guidelines set by the World Health Organization, the maximum tolerable daily intake of ZEN is established at 0.50 μg/kg (10). These regulations are essential for ensuring food safety and protecting public health.

In view of technology, elimination of this mycotoxin mainly include physical, chemical and biological techniques (11). Physical approaches consist of adsorption, high-temperature treatment, irradiation, etc. (11, 12). However, these methods have high requirements on equipment, large energy consumption, and are generally suitable for materials with light mildew and large particles (12, 13). The chemical methods involve treating ZEN-contaminated materials with acid, alkali, ammonia and strong oxidizing reagents (14, 15). Nevertheless, chemical reagents tend to destroy toxins structures and cause reagent residue (15). In contrast, microorganism methods have advantages of mild reaction conditions, low energy consumption, environmental friendliness, less nutrients destruction, strong specificity (11, 16).

Biodegradation and biosorption thereby have been the most frequent methods, since some of microbial species could be resource of food industrialization (17). Biodegradation is using microorganisms or enzymes to specifically degrade toxins, non-toxic or low-toxic degradation products (18, 19). However, during mycotoxins biodegradation, harmful and unknown by-products might be created (18). By contrast, microbial materials combine with toxins to form adsorption complexes, which are then removed by filtration, etc., usually suitable for liquid systems (20). Furthermore, thalle-toxin complex structure is relatively stable, only a very small number of complexes will occur desorption phenomenon after repeated washing, which is a limited reversible process (21, 22). The microorganisms such as lactic acid bacteria and yeast showed high adsorption capacity of mycotoxins due to their complete cell wall structure (23, 24). Besides potent of species, it is also influenced by temperature, pH, solutions, and dry methods (25, 26). Therefore, biosorption needs further study. Nevertheless, many studies on mycotoxins binding used microbiological cells in solution, making it much difficult to access their applicability in food. A potential solution to the problem is to cells and use them as food additives to reduce the level of mycotoxins exposure, without affecting properties of the final product.

During further investigation, besides monitoring mycotoxin removal, it is very important to develop applicable methods to detect residual toxins. According to published reports, immunochemical or other analytical methods like HPLC are possible applicable methods to monitor bioadsorption (27, 28).

*Geotrichum candidum*, a strain isolated from traditional Kefir in China, contains polysaccharides in cell wall which provide various active groups for hazardous material adsorption (29–31). What’s more, preliminary research indicated that cells in varying solutions and drying mode states exhibit distinct ZEN adsorption capabilities.

In the above-mentioned context, the primary objective of this study was to assess the effectiveness and kinetics of ZEN removal by *G. candidum* LG-8 and its fragments, with a specific focus on the influence of incubation solutions and cell-drying methods. In addition, a visual analysis of the dried strain fragment’s morphology before and after exposure to ZEN was conducted, using scanning electron microscope (SEM). The findings from our investigations will contribute to the development of novel approaches for ZEN adsorption in the food industry and the treatment of contaminated water.

## 2 Materials and methods

### 2.1 *Geotrichum candidum* LG-8 and culture conditions

*G. candidum* LG-8 is a yeast-like strain isolated from kefir in Tibet, China. The LG-8 strain was maintained and subcultured periodically three times on yeast extract peptone dextrose medium (YPD, 2% glucose, 2% peptone and 1% yeast extract) at 30°C and 180 rpm for 24 h.

### 2.2 Preparation of solutions and dry cell fragments

Before the above-mentioned washing performance, both water (Wahaha, China) and 0.15 mol/L NaCl solution were sterilized at 121°C for 20 min, while 80% (v/v) methanol was filtered through a membrane filter (0.22 μm, nylon 6).

Before 55°C-drying and lyophilization (freeze-drying) process, YPD broth containing *G. candidum* LG-8 were centrifuged at 940 × g and 4°C for 10 min. Supernatant was completely removed and the LG-8 pellets were then washed with ultra-pure water. After this process, an additional same centrifugation was taken to remove the residual water in sediment containing LG-8 pellets. 55°C-drying process was then performed by drying the collected LG-8 pellets at 55°C to a constant weight. Lyophilization process started with freezing LG-8 at −20°C for 24 h. Then, the cells were placed in a lyophilizer (initial temperature of −20°C), and the temperature was gradually decreased to a final temperature of −40°C. Both the two samples of cells were crushed.

### 2.3 Adsorption assay of ZEN using LG-8 cells and fragments

Standard ZEN (Sigma) was dissolved in methanol to make a stock solution at 1 mg/mL. This solution was added to commercial Wahaha pure water, methanol or saline, resulting in 1 μg/mL of ZEN-contaminated solvents.

The solvent tests were performed in falcon tubes containing 5 mL of ZEN-contaminated water, saline or methanol. Fresh LG-8 biomass ranging from 0.01 mg to 0.15 mg (6.50 g of fresh LG-8 cells equaled 1.00 g of LG-8 cells dried at 55°C) were suspended in these solutions. The cell/ZEN suspension was aerobically incubated at 30°C and 180 rpm for 24 h.

The drying method experiments were performed with adding various weights (ranging from 7.5 to 200 mg) of 55°C-drying or freeze-drying LG-8 fragments to 5 mL of ZEN-contaminated water. The mixture was agitated under the same condition to freeze-drying experiments in a shaker.

After 24 h, cell/ZEN solutions were centrifuged at 940 × g for 10 min. Subsequently, ZEN concentration in the supernatant was determined using High Performance Liquid Chromatography (HPLC). ZEN-contaminated solution without *G. candidum* LG-8 was also incubated as a control.

### 2.4 Stability of ZEN/LG-8 fragments complexes at various pH levels

To determine complexes stability of ZEN/LG-8 fragments, fragments bound ZEN were sequentially washed with buffer solutions at pH 7, 3 and 8 simulating human gastrointestinal tract conditions. Firstly, under simulated oral digestion conditions, LG-8 fragments were mixed with 10 mL of 0.1 M phosphate buffer (pH 7), incubated at 37°C and 180 rpm for 5 min. The first supernatants were collected by centrifuging the cells at 940 × g and 4°C for 10 min to determine the amount of released ZEN. Secondly, fragment sediments were resuspended with 10 mL of 0.1 M phosphate buffer (pH 3) at 37°C by shaking at 180 rpm for 2 h to mimic stomach conditions. Afterwards, the second supernatants were collected as above-mentioned centrifugation process. Finally, fragments were resuspended with 10 mL of 0.1 M phosphate buffer (pH 8) to simulate small intestine conditions. The same incubation for 2 h and centrifuge process was repeated to obtain the third supernatants. The amount of ZEN in all three supernatants was measured using high-performance liquid chromatography (HPLC). The released percentage of ZEN was calculated using the following Eq. (1):


(1) $$Y=\frac{A}{B}\times 100\%$$

*Y*: Released percentage of ZEN; *A*: peak area of ZEN released from the ZEN/LG-8 complexes into the supernatant; *B*: peak area of ZEN in the ZEN/LG-8 complexes.

### 2.5 Kinetic and isotherm studies

Kinetic and isotherm analysis were performed as described in above section 2.3. This isotherm model incorporates features of both the pseudo-first order and pseudo-second order models while Langmuir and Freundlich isotherms were used in this study. Two kinetics tests were finished with 1 μg/mL of ZEN-contaminated water and 20 g/L of 55°C-drying fragments. The biosorbent was separated from the solution by centrifugation (940 × g for 10 min) at predetermined time intervals (1–24 h). Then, the residual ZEN concentration in water was analyzed by HPLC. The adsorption isotherm was obtained for a constant biomass concentration and different concentrations of ZEN at 30°C.

The pseudo-first (2) and pseudo-second (3) order rate equations were expressed in a linear form as:


(2) $$\ln\;\left(Q_{e}-Q_{t}\right)=\ln Q_{e}-\frac{K_{1}t}{2.303}$$

(3) $$\frac{t}{Q_{t}}=\frac{1}{K_{2}Q_{e}^{2}}+\frac{t}{Q_{e}}$$

*Q*_e_: uptake of ZEN at equilibrium; *Q*_t_: uptake of ZEN at time t; *t*: the contact time; *K*_1_: the pseudo-first order rate constant; *K*_2_: the pseudo-second order rate constant.

The Langmuir (4) and Freundlich (5) isotherms were linearized by the following equations:


(4) $$\frac{C_{e}}{Q_{e}}=\frac{C_{e}}{Q_{m}}+\frac{1}{K_{L}Q_{m}}$$

(5) $$\ln\;Q_{e}=\frac{\ln C_{e}}{n}+\ln\;K_{F}$$

*C*_e_: ZEN concentration at equilibrium; *Q*_e_: ZEN uptake at equilibrium; *Q*_m_: maximum ZEN uptake; *K*_L_: the Langmuir adsorbent constant; *K*_F_: the Freundlich adsorbent constant.

### 2.6 ZEN analysis

The HPLC system used in this study was equipped with the following components: SIL-20A autosampler, DGU-20A5R pump, RF-20A fluorescence detector, and CTO-20A column oven. An analytical C18 reversed-phase YMC-Pack ODS-AQ column was utilized. The mobile phase, consisting of methanol, water and acetonitrile (in a ratio of 65:35:1, v/v), was passed through a membrane filter (0.22 μm), degassed, and pumped at a rate of 1.0 mL/min. For detection, a 50 μL aliquot was employed. The excitation wavelength was set at 280 nm, and the emission wavelength was 460 nm.

To determine the retention times under the working conditions, standard ZEN solutions at concentrations ranging from 0.5 × 10^−2^ μg/mL to 1.0 μg/mL were injected. The limit of detection (LOD) was determined to be 32.5 × 10^−4^ ng/mL, and the retention time was approximately 10.5 min.

The adsorption efficiency of ZEN by LG-8 was calculated using the following Eq. (6):


(6) $$Y=\frac{\left(C_{1}-C_{2}\right)}{C_{1}}\times 100\%$$

*Y*: efficiency of ZEN adsorption; *C*_1_: the peak area of LG-8 in initial supernatant; *C*_2_: the peak area of ZEN in final supernatant.

### 2.7 SEM analysis

SEM was applied to investigate morphological characteristics of cell fragments dried using different methods. The fragments were dried using a critical point device with CO_2_. After being placed on coverslips and coated with gold particles by sputtering, the samples were then visualized via SEM (S-3000N, Hitachi, Japan).

### 2.8 Statistical analysis

Origin (Version 2018), SPSS Statistics (Version 19) and Excel (Version 2010) were applied to statistical analysis. Triplicates were prepared for all experiments. Data are expressed as the mean ± standard deviation.

## 3 Results and discussion

### 3.1 Effects of solutions

**Figure 1:**
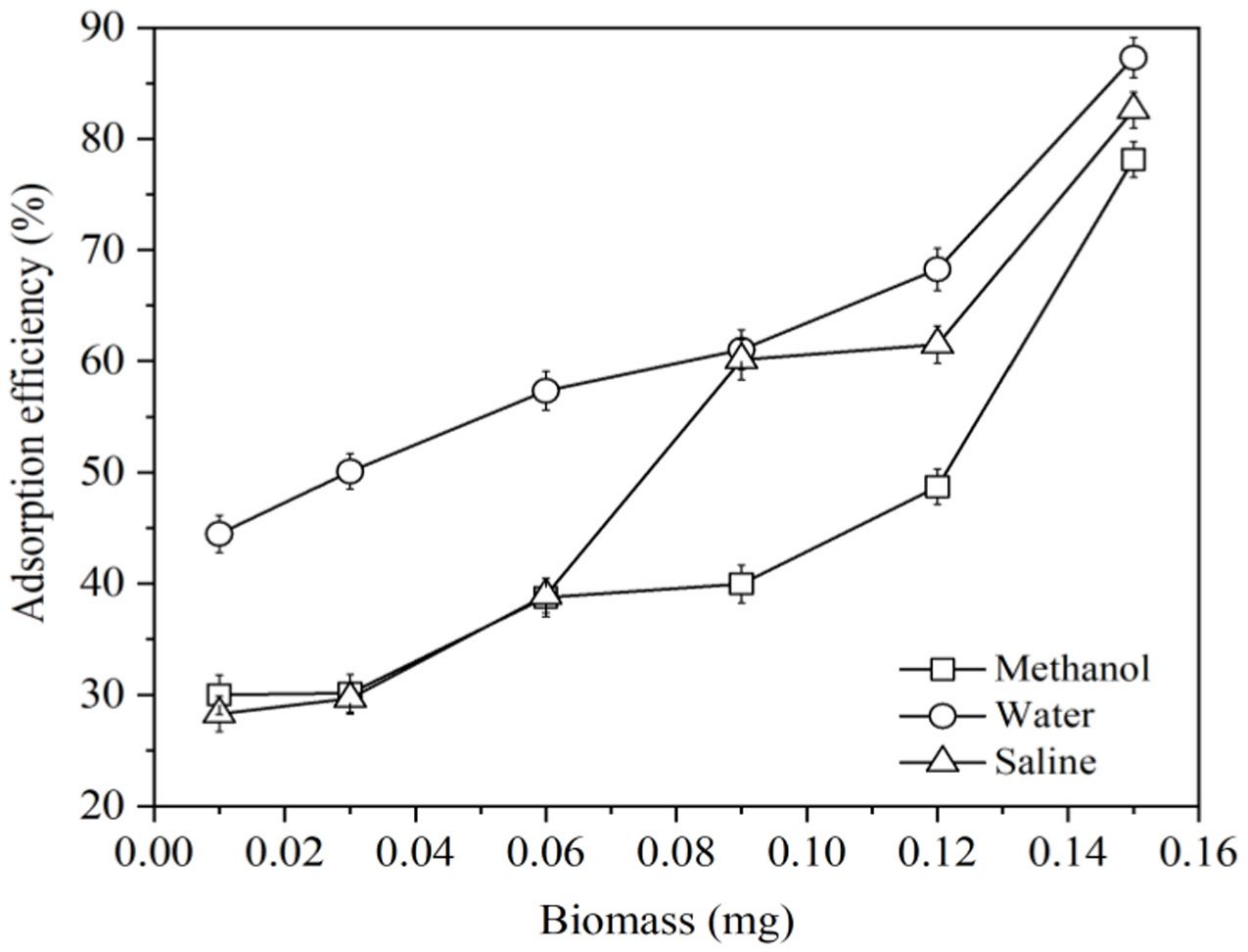
Adsorption efficiency of ZEN by *G. candidum* LG-8 cells in saline, water and methanol.

Liquid foods include water, saline, methanol, etc. It is worth to study the ability of LG-8 cell to adsorb ZEN in these solutions. This work could also help select suitable solvent for subsequent studies. In Figure 1, cells in water and saline displayed stronger ability to bind ZEN than those in methanol. At a dose of 0.15 mg, all three groups neutralized around 80% of total ZEN, which were 87.31%, 82.59% and 78.14%, respectively. Notably, 0.01 mg of LG-8 in water adsorbed a significant 44% of ZEN, about twice as much as in saline and methanol. In addition, groups of water, saline and methanol decreased in order, highlighting that LG-8’ZEN adsorption ability is influenced by the solvent used. Aqueous solutions significantly enhance the efficiency of ZEN adsorption by LG-8. Some authors proved that active groups of cell wall bind combine with substances through covalent binding, and enzymes degrade substances by regulating cell function and releasing metabolites (27, 32). Those process are generally influenced by the type of solution and its anions (21, 27).

As organic solvents are toxic to yeast and can damage cell membranes, jeopardizing the structural and functional integrity of cells. Charges of sodium ions and chloride ions may competitively bind hazardous materials to the active sites on the surface of yeast cells (29). In addition to possibility of cell rupture, ultra-pure water does not have the above two destructive forces. These factors could be considered responsible ZEN binding capacity of LG-8 in water is higher than that in saline and methanol. Therefore, water is applied as solution to study dried LG-8 fragments to adsorb ZEN.

### 3.2 Effects of drying modes

**Figure 2:**
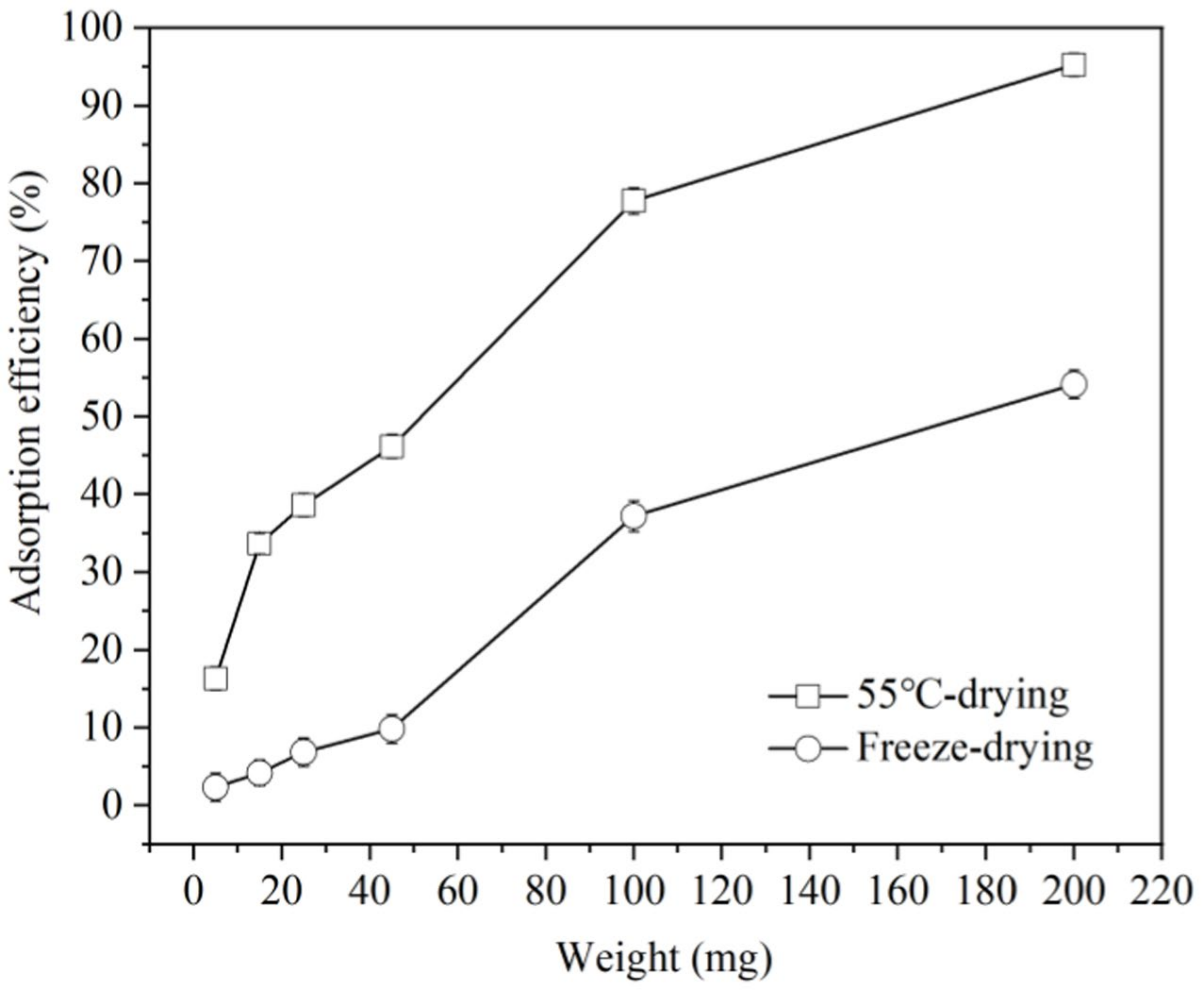
Adsorption efficiency of ZEN by *G. candidum* LG-8 fragments with 55°C-drying and freeze-drying modes.

Lyophilization, 55°C-drying and spray-drying methods are common at industrial and laboratory level. But considering the convenience and cost of equipment, lyophilization and 55°C-drying modes were used to study the impact of them on ZEN adsorption by LG-8 fragments in water. The results in Figure 2 revealed that ZEN adsorption efficiency of LG-8 dried at 55°C increased from 16% to 95% with a proportional increase in biomass weight. Notably, it had not reached equilibrium, surpassing the efficiency of lyophilized LG-8 which is less than 55% at maximum biomass weight. These findings suggest that ZEN adsorption benefits from the 55°C-drying mode, with an efficiency even higher than previous research by some authors (33). Furthermore, ZEN adsorption capacity of 55°C-drying LG-8 cells did not differ significantly from fresh cells in solutions. Some authors reported that chemical reagents, mircorganisms have the ability to adsorb hazardous materials, the adsorption capacity is lower than LG-8 or in the adsorption process combined with biological transformation, producing new poisons, and/or affecting product characteristics (33). Therefore, the importance of 55°C-drying mode should be re-emphasized.

Previous studies reported that dehydration is an important factor responsible for cellular damage (34). This is because water in cells contributes significantly to cell stability and its removal can lead to structural and functional integrity changes in cell membrane (34). The dehydration processed by heating is prone to thermal stress which also can damage cellular. Additionally, cells suffered more damage with the increase of dehydration temperature. Spray drying is a typical method with high temperature (34, 35). 55°C used in our study is much lower than those methods with high temperature, and LG-8 cell fragments dried at 55°C have a more prominent ability to bind ZEN and did not differ significantly from fresh cells in solution. It is advocated due to reducing loss or destruction of cell wall in response to dehydration treatments under mild temperature (35).

Unlike 55°C-dried cells, lyophilized cell fragments decreased the capacity to adsorb ZEN, and differ significantly from fresh cells in solutions. Lyophilization involves freezing cells and drying them by water sublimation. Freezing forms extra-cellular ice crystals which increase extra-cellular osmolality, result in beginning cell dehydration (36). As temperature drops, concentrations of both intracellular and extra-cellular solution increase until reaching a eutectic point (36, 37). According to studies, the ice crystals can damage structure and function of cell wall, cell membrane and DNA (37). Freeze-dried LG-8 cells differ significantly from 55°C-dried and fresh cells in water. These factors can be considered to cause a lower ZEN adsorption capacity by freeze-dried *G. candidum*. In studies, the pre-culture fresh cells and those under heat treatment also showed higher ZEN adsorption capacity than freeze-dried ones. The authors reported similar considerations and speculations.

Comparing with lyophilization, 55°C-drying process is much cheaper, besides maintains higher ability of *G. candidum* in adsorbing ZEN. Additionally, to obtain 1 g of *G. candidum*, lyophilization uses about twice as much fresh cells as 55°C drying. Therefore, 55°C-drying mode is the preferred drying condition. Such an inexpensive process can be widely used in food industry. Dried biosorbents would facilitate the transportation and storage conditions. In addtion, they could be added directly in food products through applying them to liquid products for a period of time or being used as a food additive in solid food products. Therefore, it is also worth to study and discuss LG-8/ZEN complexes at various pH and the LG-8 morphology in the following sections.

### 3.3 Stability of ZEN/LG-8 fragment complexes at various pH values

It is important to evaluate stability of ZEN/LG-8 complex to predict ZEN release during gastrointestinal passage and consequently to estimate the real binding potential of ZEN. Therefore, after adsorption assays, the collected dried fragments were assessed for complex stability in simulated vitro conditions (7, 3, and 8). Table 1 showed that all ZEN total released percentages were consistently below 2% (1.969% and 1.262%, respectively), underscoring the high stability of the ZEN/LG-8 complexes. The finding is different from those reports, who reported reversible adsorption of ZEN to microorganisms (38).

**Table 1:** Stability of the ZEN/LG-8 complexes in stimulated gastrointestinal tract (pH 7, pH 3 and pH 8).

Samples	Weight (mg)	Releasing percentage of ZEN (%)			
		pH 7	pH 3	pH 8	Total
55°C-drying	200	1.171 ± 0.02^*^	0.053 ± 0.00^*^	0.038 ± 0.01^*^	1.262
Freeze-drying	200	1.753 ± 0.02^*^	0.057 ± 0.01^*^	0.159 ± 0.01^*^	1.969

Values were the mean ± standard deviations. ^*^Values are not significantly different from control experiments (*p* < 0.05).

By 55°C-drying LG-8 fragments, percentages of ZEN loss were only 1.171%, 0.053% and 0.038% at pH 7, pH 3 and pH 8, respectively. Values are not significantly different from control experiments and among groups. Notably, the loss is also a little lower than those by freeze-drying ones. Furthermore, although the most favourable pH for ZEN/LG-8 complexes was pH 8 while pH 7 was the least retained ZEN. In this work, biomass were crushed to fragments without viable cells. It is rational that the adsorption characteristics of strain are dependent on cell wall with active groups. Effects of pH could be due to toxins and ions such as hydronium ions competitively bind to charges on the groups. The ester bonds of ZEN can be cleaved under alkaline conditions and regenerated when the pH is decreased (5). All these factors could be consider responsible for not significant differences in 3 pH environments. Petruzzi et al. (39) reported that high amounts of ZEN (80%–85%) were released after being washed 3 times within 5 min. Besides that, other reports proved that yeast integrated active cells in simulated vivo gastrointestinal conditions retained only about 30% of ZEN after 3 washes of 6 h and 5 min (38). In our case, the total washing time was 4 h and 5 min. Consequently, it is expected that, LG-8 fragments can keep ZEN for a plenty time until being excreted through faeces. Additionally, real intake levels of ZEN would be less than the amount in this study. It should be also noted that fragments instead of active cells unlikely to infect immunodeficient patients and compromise final product characteristics. In future, LG-8 will be tested *in vivo* gastrointestinal and industrial environments to confirm its potential.

### 3.4 Kinetic and isotherm studies

**Figure 3:**
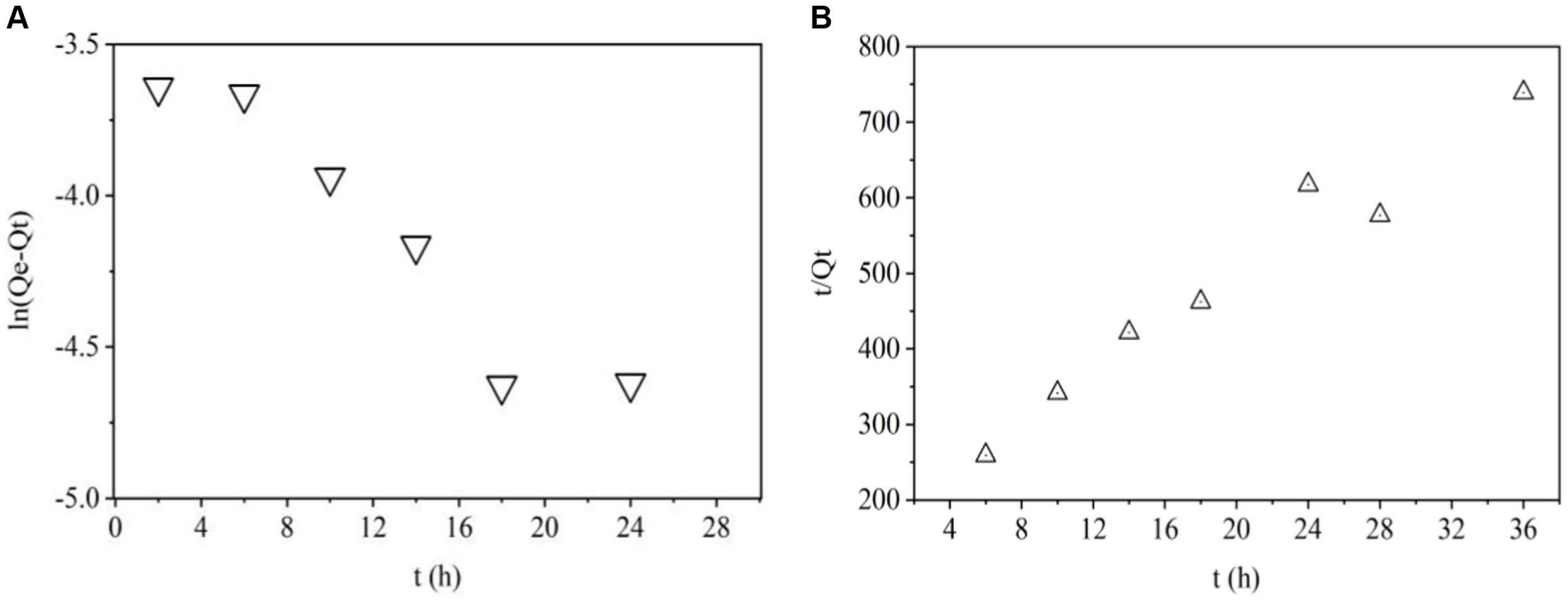
Pseudo first **(A)** and second order **(B)** kinetic models.

**Figure 4:**
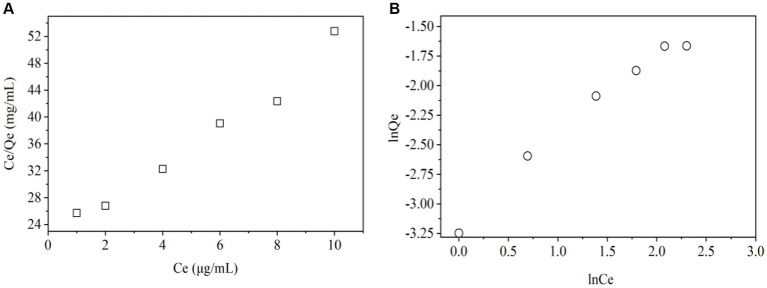
Langmuir **(A)** and Freundlich isotherm models **(B)**.

To know the adsorption mechanisms and speed of the process, it is important to study the mass transfer and chemical reactions. To do this, kinetic and isotherm were studied. Figures 3, 4 show that pseudo-first and pseudo-second order kinetic models, Langmuir and Freundlich modes have good correlation with the ZEN adsorption process.

Pseudo-first-order model is used to predict kinetic behavior of physical adsorption (40). The plots of ln (*Q*_e_ − *Q*_t_) VS *t* for this model were shown in Figure 3A. *R*^2^ value was 0.607 in Table 2 indicating that adsorption of ZEN onto LG-8 fragments did not not follow pseudo-first-order kinetic model. Moreover, it can be seen that the experimental value of *Q*_e,exp_ and theoretical *Q*_e1,cal_ value were 0.189 and 0.074, respectively. They were not closer to each other. Therefore, the pseudo-first-order model is not suitable for modeling adsorption of ZEN onto *G. candidum* fragments.

**Table 2:** Pseudo first and second order kinetic models.

ZEN concentration (μg/mL)	*Q*_e,exp_ (μg/mg)	Pseudo first order			Pseudo second order		
		*K*_1_	*Q*_e1,cal_	*R* ^2^	*K*_2_	*Q*_e2,cal_	*R* ^2^
1	0.189	0.343	0.074	0.607	0.024	0.107	0.945

Pseudo-second-order model is used to predict kinetic behavior of chemical adsorption as a rate-controlling step (40). The linear plots of *t*/*Q*_t_ VS *t* for adsorption of ZEN onto LG-8 fragments from 4 h to 36 h were shown in Figure 3B. *R*^2^ value was 0.945, which was very high. Additionally, Table 2 showed that the theoretical *Q*_e2,cal_ value was 0.107, closer to the experimental *Q*_e,exp_ value (0.189). Therefore, the pseudo-second-order kinetic model better fitted the biosorption of ZEN onto LG-8 fragments comparing with pseudo-first-order model.

The Langmuir isotherm model (Figure 4A and Table 3) indicates the relationship between the amount (μg) of ZEN adsorbed per unit mass (mg) of LG-8 fragments against the concentration of ZEN residual in water (μg/L). The *R*^2^ value was 0.998, indicating that binding of the toxin onto LG-8 fragments fitted well the Langmuir isotherm model. In another word, adsorption of ZEN onto LG-8 occurred at functional binding sites on the surface of fragments and it is monolayer adsorption. Values of *Q*_m_ and *K*_L_ were 0.342 and 7.314, respectively. Comparing with other reports, adsorption capacity of *G. candidum* LG-8 fragments for ZEN is similar to that of the majority of other integrated live and dead biomass mentioned (40, 41).

**Table 3:** Langmuir and Freundlich isotherm models.

*Q*_e,exp_ (μg/mg)	Langmuir			Freundlich		
	*K*_L_	*Q*_m_ (μg/mg)	*R* ^2^	1/*n*	*K*_F_	*R* ^2^
0.189	7.314	0.342	0.998	0.803	0.043	0.980

The Freundlich isotherm model (Figure 4B and Table 3) assumes heterogeneous surfaces with active sites relating different energy. 1/*n* of this model is an empirical parameter related to the adsorption intensity. It varies with the heterogeneity of binding material. In Table 3, the values of 1/*n* and *K*_F_ were 0.803 and 0.043, respectively. The 1/*n* value was between 0 and 1 indicating that the adsorption of ZEN onto LG-8 fragments was favourable. However, the *R*^2^ value was 0.980. It indicates that Freundlich isotherm model was not adequately able to describe the relationship between absorption amounts of ZEN and its equilibrium concentration in water. Therefore, Langmuir isotherm model is suitable for the equilibrium data since it shows higher *R*^2^ value.

### 3.5 SEM analysis of cell fragments prepared by different drying modes

**Figure 5:**
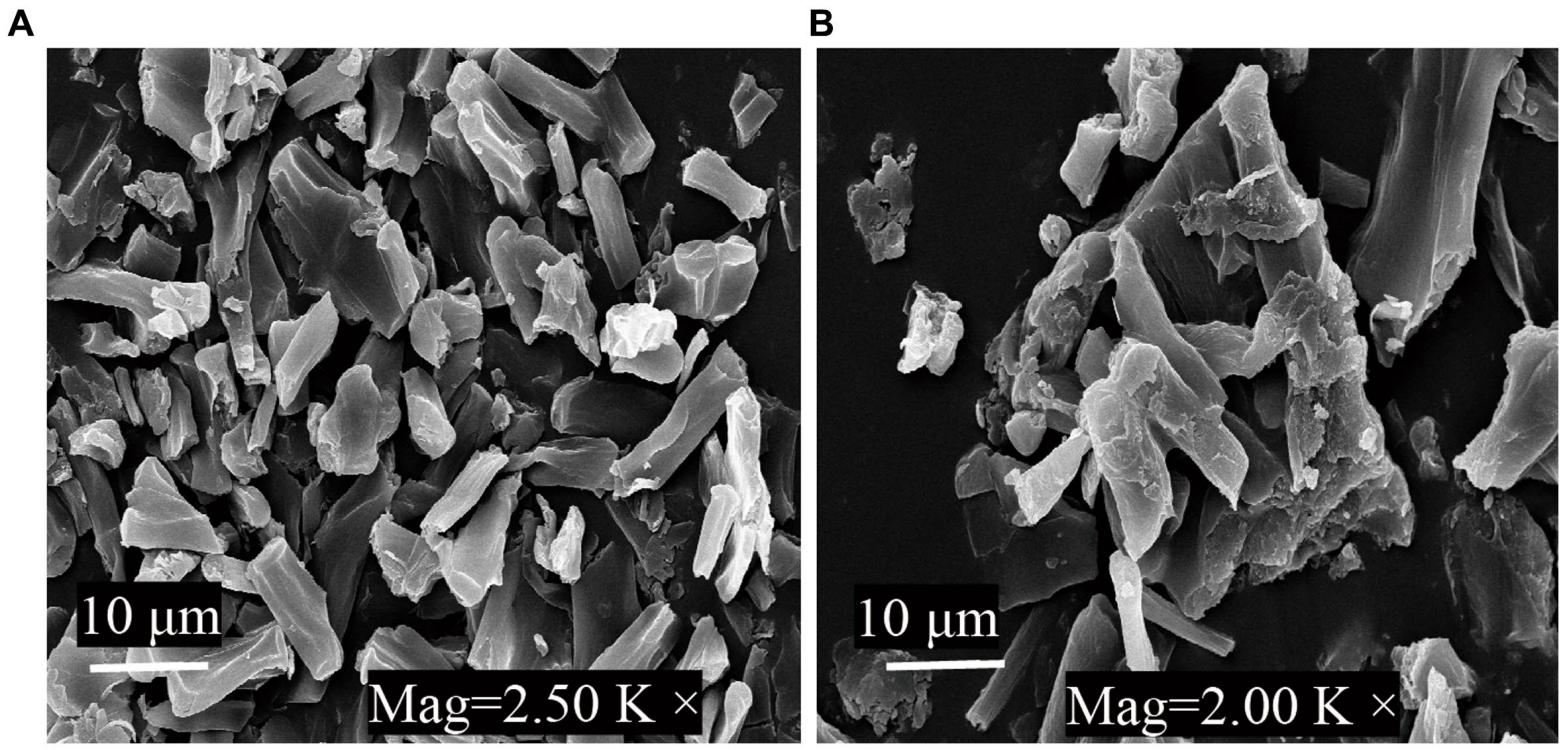
SEM micrographs of *G. candidum* LG-8 fragments dried at 55°C **(A)** and by lyophilization **(B)**.

Figure 5 shows SEM images of *G. candidum* LG-8 cell fragments dried by 55°C and lyophilization. In Figure 5A, 55°C-dried samples were viewed at a magnification of 2.5K×, generally existed in the form of fragments without significant aggregation. In contrast, those dried by lyophilization (Figure 5B) displayed fragmentation and aggregation. Fragments mean a reduction in the number of intact cells. What’s more, it has been proved in section 3.2 that both cells dehydrated through 55°C-drying and lyophilization could bind ZEN. SEM analysis, together with such results indicated that inactive *G. candidum* LG-8 such as its fragments retained the function of binding toxins. Similar studies have been verified by Shang et al. (41). The authors found that integrity of cell wall plays a crucial role in adsorbing toxins by viable and nonviable cells. They also verified that purified fragments were able to bind toxins. Loss or destruction of cell wall decreased binding capacity of cells.

Similar phenomenon was observed in ZEN binding process by freeze-dried *G. candidum* LG-8. Besides fragmentation, aggregation observed on surface of those cells was considered to contribute to reduction in binding toxins. During lyophilization, large ice crystals can damage cell structures (30, 32). In addition, increased extracellular osmolality tends to lead to leakage of cellular contents. All these factors could be consider to contribute to aggregation. This emphasizes the importance of cell fragment independence, because they can be inactivated, but after the drying process, they have to retain ability to provide surface sites for ZEN adsorption. Thus, alternatives to lyophilization to cause less aggregation of cells and cell fragments would be the use of 55°C drying method. High capacity of LG-8 fragments in this study provide possibility of their application as food additives to reduce the exposure level to ZEN, without compromising the final food product characteristics. Therefore, drying with hot air (55°C) is a practical alternative to lyophilization.

## 4 Conclusion

The study showed that ZEN adsorption efficiency by 0.15 mg of LG-8 cells in water, saline and methanol contaminated with 5 mL of ZEN (1 μg/mL) were 87.31%, 82.59% and 78.14%, respectively. In water, ZEN bound by 100 mg of those cell’s fragments dried at 55°C was 77.74%, higher than that of fragments dried by lyophilization. ZEN/LG-8 fragments were quite stable in simulated vitro gastrointestinal conditions (pH3, pH7, pH8), which totally released 1.969% and 1.262% ZEN. SEM results showed more aggregation among LG-8 fragments dried by lyophilization than those dried at 55°C. It is considered that destruction of the cell wall caused by ice crystals formed during the freezing process contributed to decrease of adsorption efficiency. Langmuir and Freundlich models were applied to describe the biosorption process. *K*_L_, *Q*_m_ and *R*^2^ of Langmuir isotherm were 7.314, 0.342 and 0.998. It showed that this model fitted the equilibrium data better than Freundlich isotherm. Pseudo-first-order and pseudo-second-order kinetic models were also used to test the experimental data. *K*_1_, *Q*_e1,cal_ and *R*^2^ of the model were 0.343, 0.074 and 0.607. The results showed that the ZEN biosorption process followed well this kinetic model. The kinetic and isotherm results help conclude that ZEN was bound to sites on the surface of fragments and the process was a favourable monolayer adsorption. Thus, our conclusions support that *G. candidum* LG-8 and its 55°C-drying fragments can be potential ZEN biosorbents.

## Key contribution

*Geotrichum candidum* LG-8 fragments can remove more than 90% of ZEN from water, which could be considered to instead of its dead and live cells as a potential scavenger for ZEN, reducing its impact on product quality and immune deficiency patients.

## Data availability statement

The original contributions presented in the study are included in the article/supplementary material, further inquiries can be directed to the corresponding authors.

## Author contributions

FJ: Investigation, Writing – original draft, Data curation. XC: Investigation, Writing – original draft, Data curation. SS: Investigation, Writing – original draft, Software. GJ: Resources, Writing – original draft. MD: Funding acquisition, Resources, Writing – original draft, Conceptualization, Methodology, Project administration, Supervision. LM: Funding acquisition, Project administration, Resources, Supervision, Validation, Visualization, Writing – review & editing, Conceptualization, Formal analysis, Investigation, Methodology.
